# Screening for Susceptibility-Related Factors and Biomarkers of Xianling Gubao Capsule-Induced Liver Injury

**DOI:** 10.3389/fphar.2020.00810

**Published:** 2020-05-29

**Authors:** Chun-yu Li, Ming Niu, Ya-lei Liu, Jin-fa Tang, Wei Chen, Geng Qian, Ming-yu Zhang, Ya-fei Shi, Jun-zhi Lin, Xing-jie Li, Rui-sheng Li, Xiao-he Xiao, Guo-hui Li, Jia-bo Wang

**Affiliations:** ^1^National Cancer Center, National Clinical Research Center for Cancer, Cancer Hospital, Chinese Academy of Medical Sciences and Peking Union Medical College, Beijing, China; ^2^China Military Institute of Chinese Medicine, Fifth Medical Center of Chinese PLA General Hospital, Beijing, China; ^3^The First Affiliated Hospital of Henan University of Chinese Medicine, Zhengzhou, China; ^4^Central Laboratory, Hospital of Chengdu University of Traditional Chinese Medicine, Chengdu, China; ^5^Research Center for Clinical and Translational Medicine, Fifth Medical Center of Chinese PLA General Hospital, Beijing, China

**Keywords:** Xianling Gubao capsule, idiosyncratic drug-induced liver injury, susceptibility-related factors, metabolomics, biomarkers

## Abstract

Although increasing reports from the literature on herbal-related hepatotoxicity, the identification of susceptibility-related factors and biomarkers remains challenging due to idiosyncratic drug-induced liver injury (IDILI). As a well-known Chinese medicine prescription, Xianling Gubao Capsule (XLGB) has attracted great attention due to reports of potential liver toxicity. But the mechanism behind it is difficult to determine. In this paper, we found that XLGB-induced liver injury belongs to IDILI through the analysis of clinical liver injury cases. In toxicological experiment assessment, co-exposure to XLGB and non-toxic dose of lipopolysaccharide (LPS) could cause evident liver injury as manifested by significantly increased plasma alanine aminotransferase activity and obvious liver histological damage. However, it failed to induce observable liver injury in normal rats, suggesting that mild immune stress may be a susceptibility factor for XLGB-induced idiosyncratic liver injury. Furthermore, plasma cytokines were determined and 15 cytokines (such as IL-1β, IFN-γ, and MIP-2α etc) were acquired by receiver operating characteristic (ROC) curves analysis. The expression of these 15 cytokines in LPS group was significantly up-regulated in contrast to the normal group. Meanwhile, the metabolomics profile showed that mild immune stress caused metabolic reprogramming, including sphingolipid metabolism, phenylalanine metabolism, and glycerophospholipid metabolism. 8 potential biomarkers (such as sphinganine, glycerophosphoethanolamine, and phenylalanine etc.) were identified by correlation analysis. Therefore, these results suggested that intracellular metabolism and immune changes induced by mild immune stress may be important susceptibility mechanisms for XLGB IDILI.

## Introduction

Recently, with a deep belief that traditional Chinese medicines (TCM) are safe because they are “natural,” TCM and related products are applied more and more widely in the world, especially in the fields of tonics, health products, and food (Luo et al., 2019). However, TCM-related hepatotoxicity issues have also become increasingly prominent, causing widespread concern among researchers and drug regulatory authorities. Xianling Gubao (XLGB) is extensively applied to treat osteoporosis, osteoarthritis, menopausal syndrome, aseptic bone necrosis and bone fracture for about 20 years, and has definite therapeutic effect (Cheng et al., 2013). However, many cases of liver injury associated with XLGB have emerged in recent years. The China Food and Drug Administration (CFDA) has also warned of the risk of liver damage from XLGB in 2016.

The formulation of XLGB consists of six herbs: Epimedii folium (*Epimedium brevicornu* Maxim., *Epimedium koreanum* Nakai, *Epimedium pubescens* Maxim., *Epimedium sagittatum* (Siebold & Zucc.) Maxim., or *Epimedium wushanense* T.S.Ying), Psoraleae fructus (*Cullen corylifolium* (L.) Medik.), Dipsaci radix (*Dipsacus asperoides* C.Y.Cheng & T.M.Ai or *Dipsacus inermis* Wall.), Salvia miltiorrhiza radix et rhizoma (*Salvia miltiorrhiza* Bunge), Anemarrhenae rhizoma (*Anemarrhena asphodeloides* Bunge), and Rehmanniae radix preprarata (*Rehmannia glutinosa* (Gaertn.) DC.) (Guan et al., 2011). No toxic herbs were found in the routine recipe. Moreover, no obvious adverse effects or toxicity developed in ovariectomized rats administrated XLGB at dosages 1000 mg/kg for 26 weeks (Cheng et al., 2013) or 1200 mg/kg/day for 12 months and up to 1800 mg/kg/day for 26 weeks (equivalent to six times the daily-recommended dose) (Wu et al., 2017). The safety and effectiveness of XLGB in treating osteoporosis has been proven in a randomized, multicenter, double-blind, placebo-controlled clinical trial (Li et al., 2018). Currently, in spite of predictable liver toxicity is often found in preclinical and clinical testing for drugs, it is extremely difficult to recognize or assess Chinese herbs-related to hepatotoxicity, especially idiosyncratic liver injury. Therefore, the underlying mechanisms of XLGB-induced liver injury remain unknown. Our previous research has indicated that mild inflammation might be one of the factors related to susceptibility of Chinese herbs (He shou wu in Chinese)-induced liver injury (Li et al., 2016; Li et al., 2017). Simultaneous exposure to small doses of LPS can serve on a susceptibility-related determinant of many chemicals to intoxication, such as trovafloxacin and monocrotaline (Yee et al., 2003; Poulsen et al., 2014). A critical need exists to predict IDILI of Chinese herbs and understand the mechanisms behind it. Here, we investigated the objectivity of XLGB-induced liver injury by analyzing clinical liver injury cases and simultaneously evaluated XLGB susceptibility on previously established animal models of mild immune stress. As a follow-up, we employed a metabolomics strategy in order to explore the susceptibility related biomarkers of XLGB-induced liver toxicity from the perspective of metabolic reprogramming. The immune cytokines and differentially expressed biomarkers associated with the susceptibility factors of XLGB-induced liver injury were identified.

## Materials and Methods

### Chemicals and Reagents

Xianling Gubao Capsule (batch number 1601046) was produced by Guizhou Tongjitang Pharmaceutical Co., Ltd (Guizhou, China). Epimedin A, Epimedin B; Epimedin C, icariin, psoralen, angelicin, icarisid II and anhydroicaritin were from Chengdu Pufei De Biotechnology Co., Ltd. (Chengdu, China). Sodium pentobarbital (cat 57-33-0) and LPS [derived from Escherichia coli, 055:B5 (lot 086M4159V)] were supplied by Sigma Chemical Company. Methanol and formic acid are both HPLC grade, purchased from Fisher Chemicals (Pittsburg, PA, USA); Acetonitrile is also HPLC grade, obtained from Merck (Darmstadt, Germany); Water was purified by a Millipore's ultrapure water system (Millipore, Bedford, MA, USA). Luminex multiplex cytokine analysis kits was from R&D Systems (MN, United States). Alanine aminotransferase (ALT) and aspartate aminotransferase (AST) detection kits were acquired from Jiancheng Biological Technology, Co., Ltd (Nanjing, China).

### Data Sources of Adverse Drug Reactions Cases

A total of 55,388 cases of drug-induced liver injury were collected from the National Adverse Drug Reaction (ADR) Monitoring Database (2012–2016), of which 63 cases of XLGB-related liver injury were found and included in the study. All cases were evaluated and included using the integrated evidence chain-based identification of traditional Chinese medicine in the “Guideline for diagnosis and treatment of herb-induced liver injury” issued by the China Association of Chinese Medicine and “Technical guidelines for clinical evaluation of liver injury induced by traditional Chinese medicine” issued by National Medical Products Administration (Wang et al., 2014; Wang et al., 2016; Zhu et al., 2016b). Retrospective analysis was performed for statistical analysis in this study.

### Animals and Treatment Protocol

Male, Sprague-Dawley Rats weighing 180 to 250 g (Laboratory Animal Center of the Academy of Military Medical Sciences, License No. SYXK 2007- 004, Beijing, China) were used for this research. Rats were given continual access to water and food *ad libitum*. Animals were maintained in a 12 h light/dark cycle for one week of acclimatization. They received humane care according to the Guiding Principles for the Care and Use of Laboratory Animals of China and Institutional Animal Care, and procedures were approved by the Use Committee of 302 hospital of PLA. The powder of Xianling Gubao Capsule was suspended in 0.5% sodium carboxymethyl cellulose solution.

Evaluation of idiosyncratic liver injury was according to literature and our previously reported rat model and adjusted appropriately (Luyendyk et al., 2006; Li et al., 2015). Doses of LPS and XLGB were selected from our preliminary range-finding studies. Briefly, the animals were given normal saline or 2.8 mg/kg LPS by tail vein injection. Two hours later, 1.62 g/kg Xianling Gubao Capsule or an equivalent volume of normal saline was administered intragastrically. Rats were anesthetized (sodium pentobarbital, 50 mg/kg, ip) after six hours later. Blood was gathered from the inferior vena cava using a syringe filled with 0.38% sodium citrate. Plasma was collected after centrifugation (3,000 rpm, 10 min) and stored at 20°C until use. The left lateral liver lobe (3–4 mm) were removed and performed histopathological examination after fixing in 10% neutral buffered formalin for at least 72 h (He et al., 2018).

### Assessment of Hepatic Injury and Analysis of Cytokines in Plasma

Hepatic parenchymal cell damage is assessed by an increase in ALT and AST activity in plasma, which is determined in accordance with the procedures of the microplate assay kit. Formalin-fixed liver lobes were embedded in paraffin, cut at a 4 μm, and stained with hematoxylin and eosin. Resulting slides were coded, and evaluated by light microscopy. Plasma cytokine/chemokine profiles, including IL-18A, MIP-2, MIP-2α, Rantes, MCP-3, MCP-1, IP-10, IL-6, VEGF-α, GRO-α, IL-4, IFN-γ, IL-2, IL-13, TNF-α, IL-1β, GM-CSF, IL-5, IL-11, IL-12p70, and Eotaxin were deterimined according to the manufacturer's instructions of the Cytokine array of Luminex Assays Kit.

### Sample Handling of Metabolomics Study

The plasma samples were thawed at room temperature, and 600 µl of methanol was added to the 200 µl sample for extraction. The mixture were vortexed for 1 min and centrifuged (10,000 rpm,10 min) at 4°C. Then, the supernatants were collected and filtered through a 0.22-μm syringe filter.

### Chromatography and Mass Spectrometry Conditions

The metabolic profiling analysis was performed on a Waters I-Class UPLC system (Waters Corporation, Milford, USA). The sample sequence is random, and all samples were separated on a reverse phase HSS T3 C18 column (100 mm × 2.1 mm, 1.8 µm particle size). The column temperature was maintained at 40 °C. To ensure the stability and repeatability of the system, 10 μl of each sample was combined for quality control (QC), which was inserted and analyzed in every 10 samples. The mobile phase used 0.1% formic acid in water as solvent A and 0.1% formic acid in acetonitrile as solvent B. The ﬂow rate was 0.3 mL/min. The gradient program was as follows: 0 to 1 min, 5 to 5% B, 1 to 9 min, 5 to 40% B, 9 to 19 min, 40 to 90% B, 19 to 21 min, 90 to 100% B, 21 to 25 min, 100 to 100% B. The sample inject volume was 4 µl. During the whole experiment, all the samples were held at 4°C.

For mass spectrometry analysis, Waters Xevo G2-XS QTOF/MS with positive and negative electrospray ionization sources (ESI) was used (Waters Corporation, Manchester, UK). The data collection was controlled by the UNIFI informatics platform (Waters Corporation, Manchester, UK), and the data range was 50-1200 Da. Capillary voltages were at 2.5 and 2.2 kV in positive and negative modes, respectively. For positive mode and negative mode, the cone voltage was 40 V. The applied source temperature was 130°C. The desolvation gas flow was set to 800 L/h, and the desolvation temperature was 350°C. The high collision energy scan was set at a ramp energy scan from 10 to 55 eV, and the low collision energy scan was set at 4 eV. The scan time for each function was 0.20 s. Leucine enkephalin (100 pg/µl) was applied as the lock mass, generating reference ions in negative mode of 554.2610 and positive mode of m/z 556.2771, and introducing reference ions at 10 μl/min at by a lockspray for accurate mass acquisition.

### Multivariate Pattern Recognition Analysis and Identification of Biomarkers

UNIFI informatics platform was used to pre-process all data. Normalize the intensity of each ion in relation to the total number of ions to generate a data matrix consisting of normalized peak area, m/z value, and retention time (Wang et al., 2012). Then the data was introduced into (Umetrics, Umea, Sweden) software for principal component analysis (PCA) and orthogonal partial least squares-discriminant analysis (OPLS-DA) (Zhang et al., 2016; Huang et al., 2018). To screen variables that have a significant contribution to distinguishing potential biomarkers between the two groups (LPS vs Normal group or XLGB vs Normal group), only variables with |p (corr)| ≥ 0.5 and VIP values > 1 were chose and applied for further data analysis in the OPLS-DA model. Meanwhile, significant variables with p values <0.05 and folder changes> 2 or <0.5 were considered potential biomarkers. Biochemical databases, HMDB (http://www.hmdb.ca/) and METLIN (http://metlin.scripps.edu/) were used to identify potential biomarkers. Metabolic pathway analysis was performed by the KEGG (www.genome.jp/kegg/) pathway database, which was carried out using MetaboAnalyst 3.0 (http://www.metaboanalyst.ca/) in line with the pathway library of Rattus norvegicus (rat) (Wang et al., 2012).

### Correlation Analysis

Pearson's correlation coefficient is a statistical measure of the strength of the linear relationship between paired data (Han et al., 2018; Li et al., 2019). To quantify the association between two consecutive variables, correlation analysis, a common statistical method, was applied to analyze the relevance between concentrations of cytokines and the peak area values from the UPLC-Q-TOF/MS in this study. Then, Pearson's correlation coefficient was obtained and further used to explore important related metabolites.

### Statistical Analysis

One-way analysis of variance (ANOVA, Student's t-test) was used to assess the significant difference of potential biomarkers between groups (SPSS version 15.0; IBM: Chicago, IL, USA, 2006). P < 0.05 was considered statistically significant, and P < 0.01 was considered highly significant.

## Results

### Chemical Compositions Determination of XLGB

The chemical compositions of XLGB were determined by HPLC method. The representative HPLC chromatograms of mixed standards and XLGB were shown in **Supplementary Figures 1A, B**. The results showed that there was a roughly separation within different chromatographic peaks of XLGB. Eight major components in XLGB including Epimedin A, Epimedin B, Epimedin C, icariin, psoralen, angelicin, icarisid II and anhydroicaritin were ditermined and quantified, and the relative proportion of these compounds in the preparation is 0.058%, 0.015%, 0.012%, 0.373%, 0.155%, 0.056%, 0.058% and 4.937%, respectively.

### Clinical Features of XLGB-Induced Liver Injury

**Figure 1:**
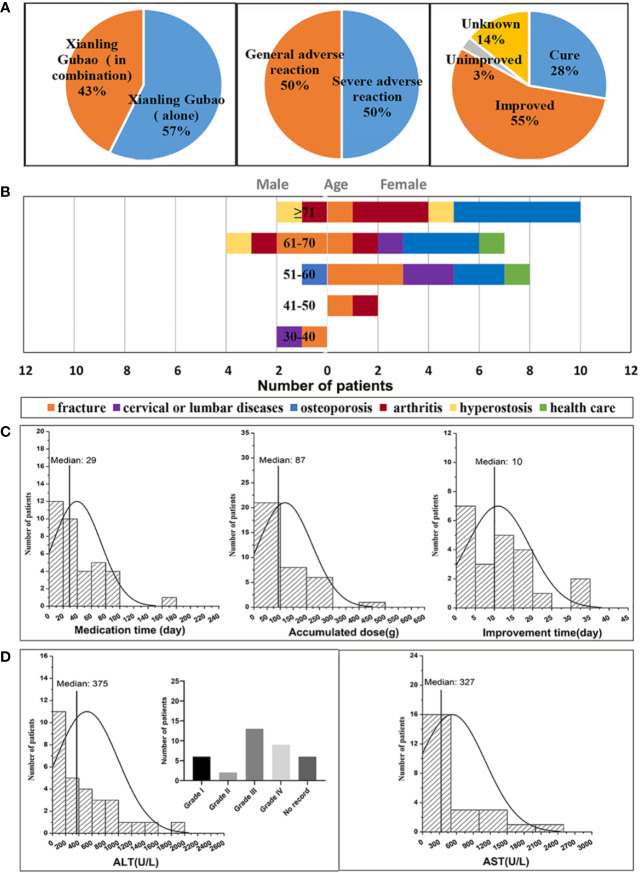
Analysis of clinical features of XLGB-induced liver injury **(A)** the incidence of XLGB-induced liver injury, drug compatibility, degree of adverse drug reaction, prognosis; **(B)** gender, age, and the purpose of medications; **(C)** Medication time, accumulated dose and improvement time; **(D)** biochemical indicators (ALT and AST).

As shown in **Figure 1A**, of the 63 cases of XLGB-induced liver injury, 36 cases were treated with XLGB alone, and 27 cases were used in combination with diclofenac sodium, omeprazole, and cisapride. Since diclofenac sodium itself is prone to liver damage, in order to reveal the objective authenticity of XLGB-induced liver injury, it is necessary to rule out the effects of other combination drugs, and only 36 cases of XLGB alone are included in the next study. Serious adverse reactions occurred in 50% of cases, and the general adverse reactions were the same. After treatment, most patients had a good prognosis, 55% of cases improved, 3% did not improve, the cure rate was 28%, and 14% of cases were unknown (**Figure 1A**).

The age distribution characteristics of liver injury cases indicated that the median age of onset was 64 years (range, 20-91 years), the ratio of males to females was 1:3.5, and the percentage is 6% in the 30-40 year-olds, 6% in the 41-50 year-olds, 25% in the 51-60 year-olds, 30% in the 5-60 year-olds, 33% in the more than 71 years old. Thus, the XLGB-induced liver injury was more common in women, and the peak age of which occurred at more than 71 years of age (**Figure 1B**). The purpose of XLGB medication included osteoporosis (30.6%), fracture (25%), arthritis (19.4%), cervical lumbar spondylosis (11.1%), hyperostosis (8.3%), and health care (5.6%), which were all in the scope of the instructions except health care (**Figure 1B**). As depicted in **Figure 1C**, the median duration of administration was 29 days. Obvious dose-to-toxic relationship was not observed between the duration of administration and the cumulative dosage. The median time of improvement after treatment drug or discontinuation was 10 days. Clinical biochemical indicators can be found in **Figure 1D**, the median of the ALT and AST levels were 10 and 9 times greater than the upper limit of the normal range (**Figure 1D**). Moreover, the higher ratio of grade 3/4 liver enzyme elevation were observed in 22/36(ALT) of patients in accordance with National Cancer Institute Common Terminology Criteria for Adverse Events (CTCAE) (Ahn et al., 2017).

### Liver Functional and Histologic Changes of XLGB

**Figure 2:**
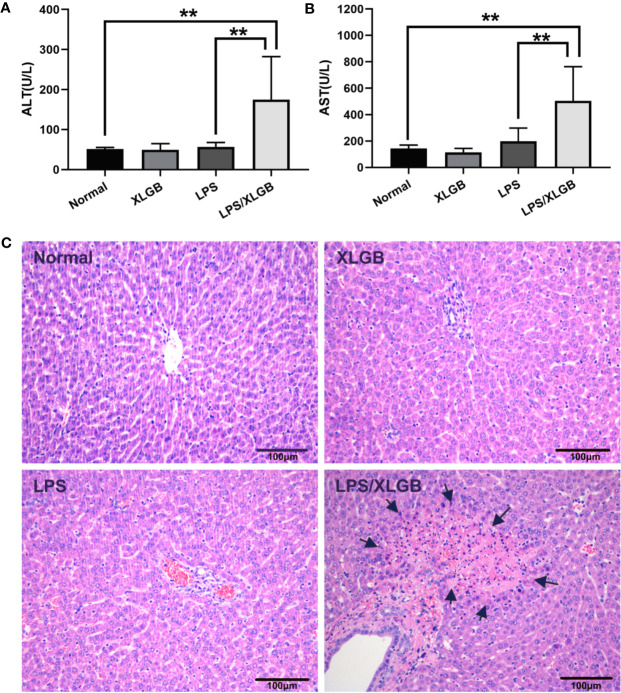
**(A**, **B)** Influence of co-treatment with LPS (2.8 mg/kg, i.v.) and XLGB (1.62 g/kg, i.g.) on plasma ALT and AST activities. **(C)** Representative microphotographs of liver sections isolated from rats. The arrows indicate multifocal midzonal necrosis of hepatocytes caused by co-treatment XLGB (1.62 g/kg, i.g.) and LPS (2.8 mg/kg, i.v.). HE staining (×200). n = 10, x ± s. ^**^P < 0.01 vs normal group or vs LPS group.

As shown in **Figures 2A, B**, compared with normal group, plasma ALT, and AST activities were not increased significantly by XLGB treatment alone (*P* > 0.05). Likewise, LPS administration did not cause increase in ALT and AST activities (*P* > 0.05). However, as marked by increased ALT and AST activities in plasma, liver injury from XLGB and LPS coexposure occurred in contrast with LPS group.

The results of histopathology displayed that no significant lesions were observed in the saline (control) and XLGB treatment groups. LPS-treated group showed evidence of mild leukocyte infiltration in portal area but no evident hepatocytes injury. However, When combined with LPS and XLGB, the severity of histologic changes was pronounced, such as multifocal midzonal necrosis of hepatocytes andmany inflammatory cell infiltration (**Figure 2C**). These data suggested that the LPS-induced inflammation activation presumably plays a crucial part in the pathogenesis of XLGB IDILI.

### Screening for Susceptibility-Related Cytokines of XLGB-Induced Liver Injury

**Figure 3:**
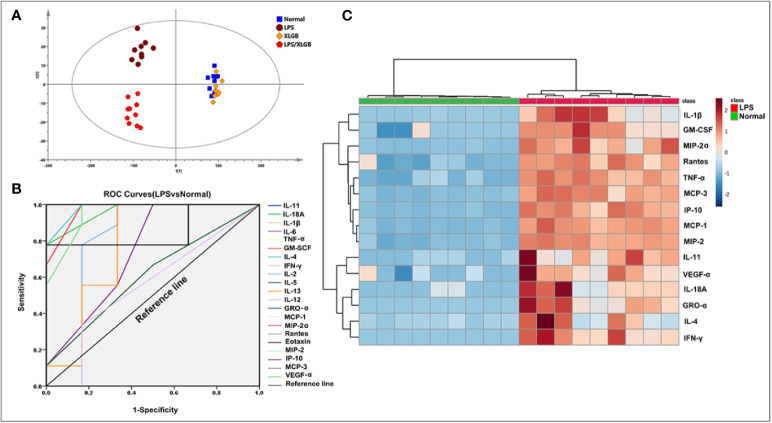
Screening for susceptibility-related cytokines of XLGB-Induced liver injury. **(A)** principal component analysis (PCA) of plasma cytokines; **(B)** receiver operating characteristic (ROC) curves of susceptibility-related to cytokines (LPS group vs normal group); **(C)** cluster analysis of susceptibility-related to cytokines (ROC > 9); the heat map colours represent concentration of cytokines relative to the minimum and maximum of all values in this analysis.

To observe possible cytokine/chemokine profiles in the normal, LPS, XLGB, and LPS/XLGB groups, the PCA analysis was performed to visualize cytokines/chemokines differences between the above four groups (**Figure 3A**). The results indicated that the LPS group and LPS/XLGB group were able to significiantly distinguished from the normal group and XLGB group. The susceptibility of XLGB-induced liver injury was therefore evident from the cytokine expression profiles. Next, The susceptibility-related cytokines of XLGB-induced liver injury were screened by a Receiver Operating Characteristic (ROC) Curve analysis (**Figure 3B**). Finally, MCP-1, IL-11, Rantes, IL-1β, IFN-γ, GRO-α, MIP-2α, IP-10, MCP-3, IL-4, MIP-2,GM-CSF, IL-18A, VEGF-α, and TNF-α were seen as potential factors (AUC > 0.9) (**Figure 3C**). Meanwhile, we performed cluster analysis of plasma-derived samples and cytokines and represented the result as a heat map. The resluts showed that there was a clear separation between the above two groups in line with cytokine expression profiles. The sample tree was divided into two clusters: cluster1: the normal group with relatively low (cold spots) cytokine expression; cluster 2: the LPS group with relatively high (hot spots) cytokine expression (**Figure 3C**).

### Metabolomic Analysis of Plasma

**Figure 4:**
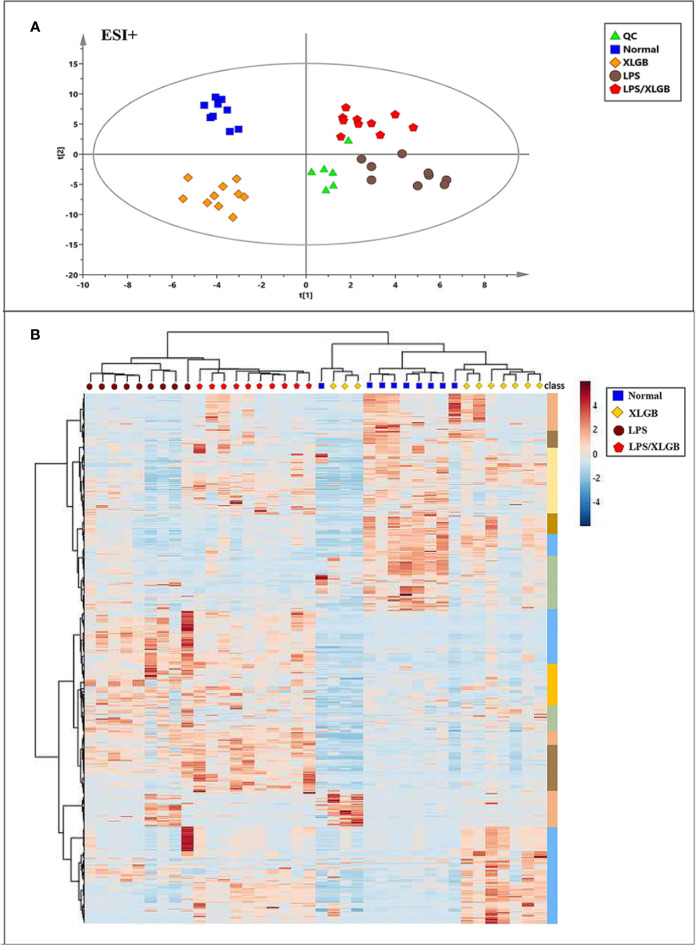
Metabolomic Profile Analysis of XLGB-induced liver injury. **(A)** PCA score plots ofof different groups in positive ESI mode; **(B)** cluster analysis of the 654 significantly changed ions among the normal, LPS, XLGB, and LPS/XLGB groups. The colors from blue to red indicate the relative contents of metabolites.

The unsupervised PCA and supervised method OPLS-DA were performed in positive and negative ion modes. **Figure 4A** showed the score plots of PCA analysis in ESI+ mode. QC samples were chosen to assess the stability and accuracy of metabolomics methods because they contain a wide range of m/z values and chemical polarities. The RSD % results showed that the within-run precision, between-run precision and stability of m/z values, retention times, and peak areas were within the error range, respectively, which verified the feasibility of the method. In addition, QC samples clustered closely in PCA score plots indicating that the LC/MS system was stable throughout the experiment. Plasma samples from normal, LPS, XLGB, and XLGB/LPS groups could be divided into different blocks by PCA, which indicated that metabolic profiles vary greatly between the four groups. In addition, there was a clear separation between the normal group and the LPS group from PCA1, indicating that the metabolic environment was significantly affected by different physiological states. Similar results in ESI- were displayed in **Supplementary Figure 2A**. Moreover, as shown in **Figure 4B** and **Supplementary Figure 2D**, the four groups (normal, LPS, XLGB, and LPS/XLGB groups) in both ESI+ and ESI-modes could also be obviously distinguished by the clustering heatmaps based on these metabolites.

**Figure 5:**
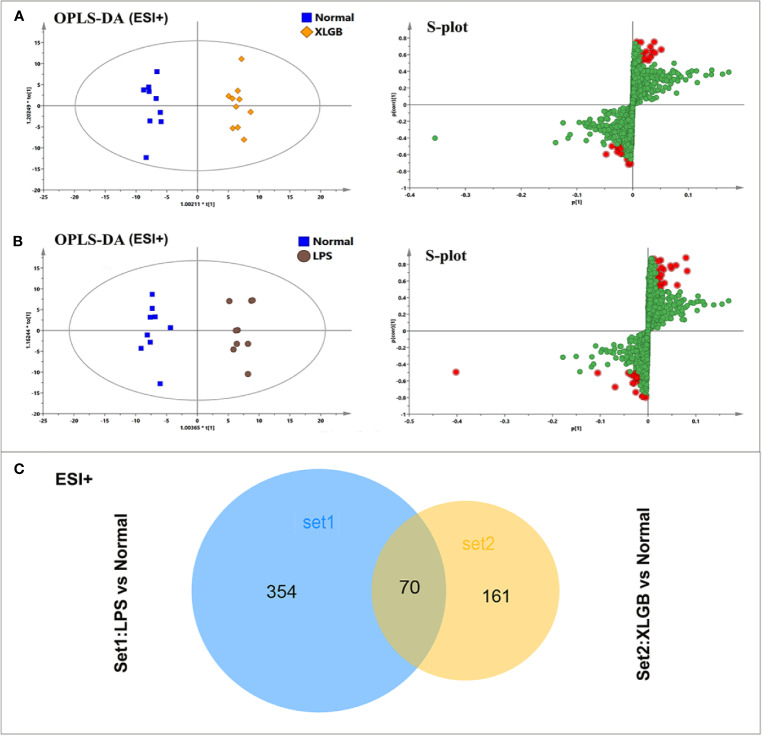
Analysis of potential biomarkers associated with susceptibility to XLGB-induced liver injury. OPLS-DA analysis of the data generated from the XLGB vs normal **(A)**, LPS vs normal **(B)** in the ESI+ mode. S-score plots constructed from the supervised OPLS analysis, the axes that are plotted in the S-plot from the predictive component are p1 vs p (corr)1, representing the magnitude and reliability respectively. Metabolite ions with VIP value >1 were marked with a red square. **(C)** The shared and unique numbers of metabolites were also visualized in Venn diagram from LPS vs normal and XLGB vs normal.

To further investigate the potential metabolites of the susceptibility-related XLGB-induced liver injury, OPLS-DA was applied to classifiy or discriminate analysis. As displayed in **Figure 5A**, and XLGB and normal groups can be clearly separated (in ESI+mode) and showed a good predictive ability with a R2Y (cum) of 0.981, R2X (cum) of 0.764, and Q2 (cum) of 0.523. Meanwhile, the LPS group could also be obviously detached from normal group. The R2Y (cum), R2X (cum), and Q2 (cum) were 0.969, 0.73, and 0.576, respectively, indicating that the OPLS-DA model was reliable. The S-plots for LPS vs normal and XLGB vs normal are shown in **Figures 5A, B**. Data analysis of ESI- mode was also performed and described in **Supplementary Figures 3A, B**. Then, the variables with a |p (corr)|≥0.5,VIP value > 1, *P* < 0.05 and fold change (FC) > 2 or <0.5 were selceted as the potential biomarkers for further analysis. Next, area-proportional 3-Venn diagrams have been used to analyze differences and similarities between differential metabolites in two different groups. The results depicted that there were 424 (LPS *vs* normal) and 231 (XLGB *vs* normal) differential ions in two independent comparisons of the ESI+ mode (**Figure 5C**), while the above comparisons were 403 and 188 in the ESI-mode, respectively (**Supplementary Figure 3C**). Therefore, the results showed that 126 differential variables were affected by the synergistic effect of LPS and XLGB, not just the individual effects of XLGB or LPS, so they could be used as potential biomarkers related to susceptibility to XLGB IDILI.

### Identification Potential Biomarkers and Enrichment Pathway

**Figure 6:**
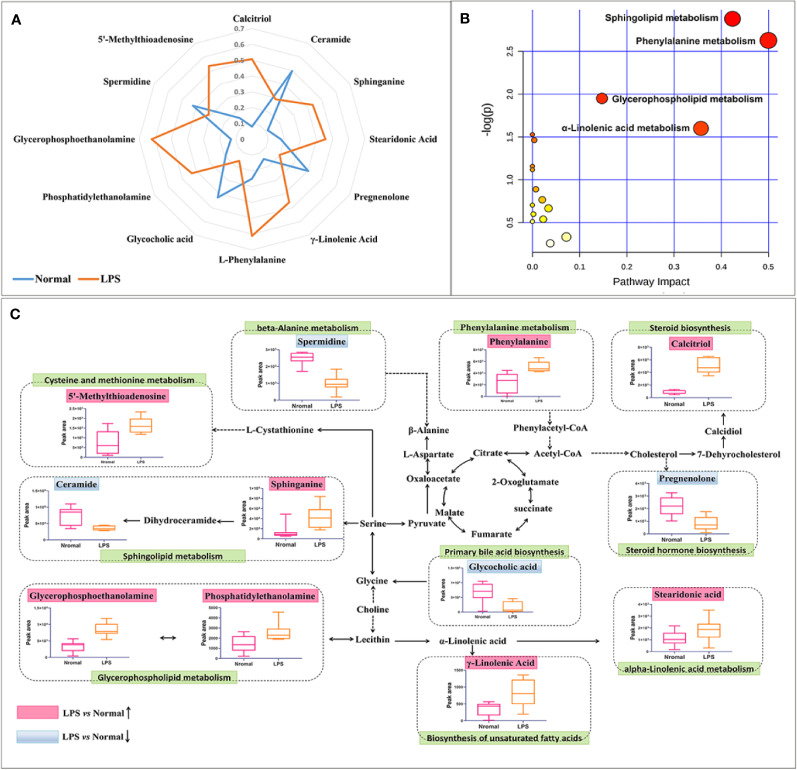
Overall metabolic profle. **(A)** spider plot of 12 metabolites of which the change in relative abundance best diferentiated between the LPS group and normal group. Blue line represents normal group, orange line represents LPS group. **(B)** Schematic diagram of the disturbed metabolic pathways. **(C)** Network map of metabolic pathways and metabolites. The notations are as follows: (↑) in red, metabolite higher in the LPS-treated group than in the normal group; (↓) in, metabolite lower in the LPS-treated group than in the normal group. The related metabolic pathways are cycled in a green box.

The acquired metabolites were combined and tentatively identified with the accurate mass charge ratio. Finally, 77 variables were considered candidates for potential biomarkers and then imported into MetaboAnalyst 3.0 and enriched in the KEGG pathway to further explore the metabolic pathways. Of these, 12 significant metabolites were obtained, including calcitriol, pregnenolone, spermidine, ceramide, sphinganine, glycerophosphoethanolamine, phenylalanine, phosphatidylethanolamine, stearidonic acid, γ-linolenic acid,glycocholic acid,5'-methylthioadenosine (**Supplementary Table 1**). In order to more intuitively compare the metabolite differences between the LPS group and the normal group, a spider diagram of the differential metabolites was constructed based on their relative abundance (**Figure 6A**). The results showed that except for pregnenolone, spermidine, ceramide, and glycocholic acid, the expression of differential metabolites in the LPS group was notably higher than that in the normal group. A schematic diagram of disturbed metabolic pathways was displayed in **Figure 4B**. Meanwhile, a complex network was constructed based on enriched metabolic pathways and the identifed differential metabolites, which contributed to contrast the differences between the LPS group and the normal group in metabolic profles (**Figure 6C**).

### Correlation Analysis of Susceptibility-Related Cytokines and Biomarkers

**Figure 7:**
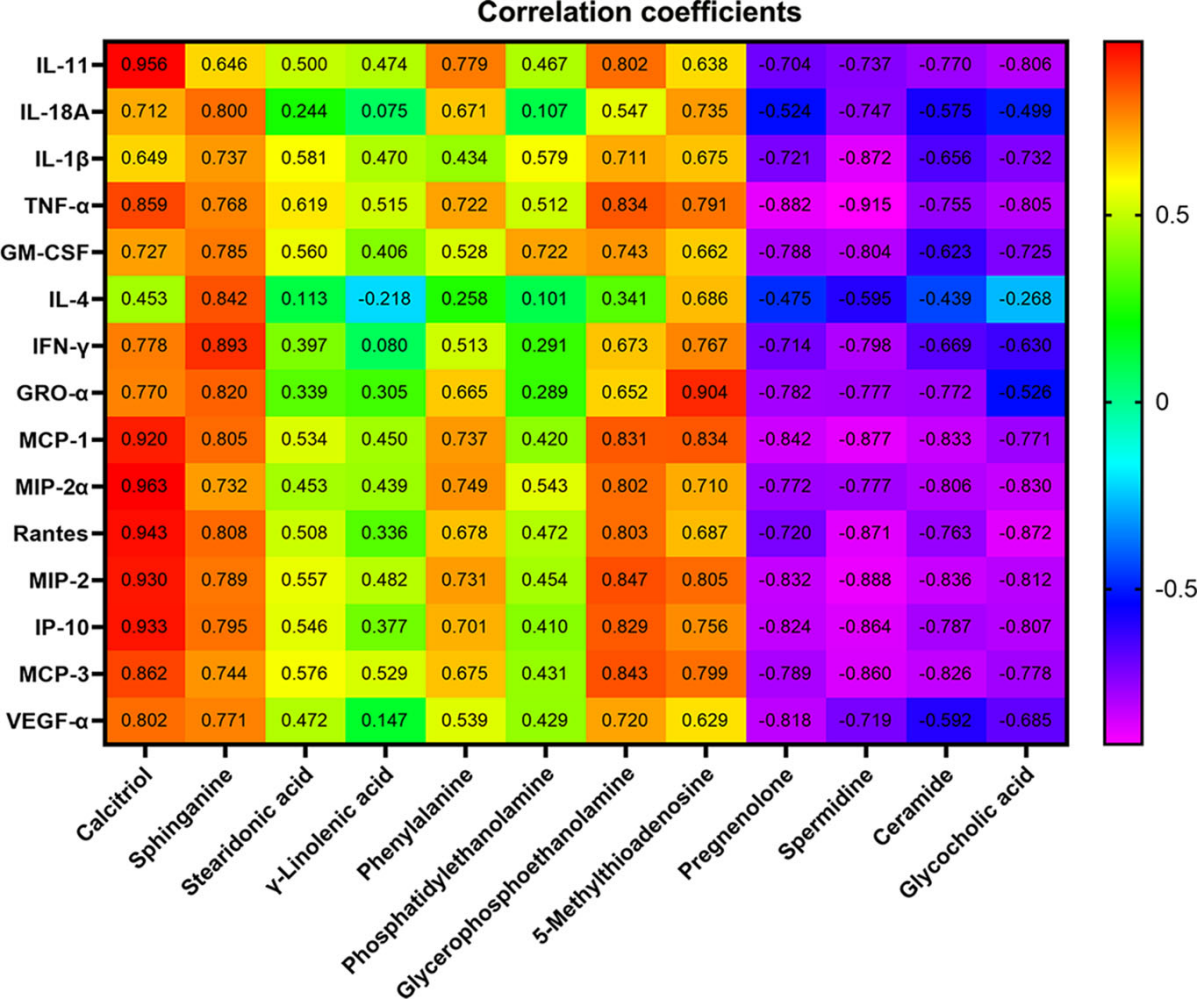
Pearson correlation coefficient of 12 metabolites and concentrations of 15 cytokines. *P* > 0.05 or *P* > 0.01(the correlation coefficient > 0.6).

To further screen for susceptibility-related metabolites of XLGB-Induced liver Injury, correlation analysis was used to evaluate metabolite-cytokine relationships between area values of 12 metabolites and concentrations of 15 cytokines. From the results depicted in **Figure 7**, the correlation coefficients demonstrated that five metabolites, sphinganine, 5'-methylthioadenosine, calcitriol, phenylalanine, and glycerophosphoethanolamine were significantly positively correlated, while pregnenolone, spermidine, ceramide, and glycocholic acid were negatively correlated. Besides, there was no correlation between γ-linolenic acid and 15 cytokines (*P* > 0.05). Compared with the normal group, the content of the five positively related metabolites increased significantly, while the content of the other four negatively related metabolites decreased. These data suggest that the change trend of the content is consistent with the positive and negative correlation of cytokines. In addition, sphinganine and 5′-methylthioadenosine were significantly correlated with 15 cytokines. Stearidonic acid and calcitriol were not correlated with 15 cytokines except for TNF-α. Specially, TNF-α was related to 9 metabolites except for phosphatidylethanolamine and γ-linolenic acid.

## Discussion

IDILI is one type of drug-related adverse reaction, which is relatively rare but may be severe or even fatal in some cases (Andrade et al., 2019). IDILI is determined by the interaction of environmental and host factors with the drug. As the significant individual differences and susceptibility of the liver to adverse drug reactions, it is still a formidable challenge to predict IDILI (Uetrecht, 2019). Despite many cases of TCM-induced hepatotoxicity have been reported, preclinical studies have not revealed and predicted their hepatotoxicity in normal experimental animals. Therefore, it is speculated that the hepatotoxicity of TCM may belong to IDILI. Our study demonstrated that the ratio of XLGB-induced liver injury in all DILI cases was very low, only 0.06% (36 in 55388), and not dose- or time-dependent by virtue of the retrospective statistics of DILI cases in the National Adverse Drug Reaction (ADR) Monitoring Database (2012-2016). Moreover, all patients took the drugs according to the instructions, and there was no overdose. Thus, XLGB-induced liver injury might be IDILI.

Currently, combined treatment of rats with endotoxin (LPS) and a model drug related to drug idiosyncrasy inpatients, led to severe hepatotoxicity in toxicological experiment assessment (Ramm et al., 2015). This model has been applied to assess IDILI of some drugs, including trovafloxacin, pyrrolizidine alkaloids, ranitidine, nefazodone, nimesulide, clarithromycin, and telithromycin successfully (Tukov et al., 2007; Guo et al., 2013; Hammad et al., 2013). In our previous work, Polygoni multiflora radix (*Reynoutria multiflora* (Thunb.) Moldenke), Epimedii folium (*Epimedium brevicornu* Maxim.), and Psoraleae fructus (*Cullen corylifolium* (L.) Medik.) could induce acute liver injury in the LPS rats, suggesting that this model could evaluate the susceptibility of the liver to hepatotoxic chemicals from TCM (Li et al., 2015). In this study, we found that a single administration of XLGB or LPS did not develop a significant liver injury phenotype, but co-administration of XLGB with LPS to rats resulted in an increase in the plasma ALT and AST levels and the severity of histologic changes. Additionally, LPS led to a slight infiltration of inflammatory cells in portal area and upregulation of cytokines such as IL-11, GRO-α, IL-1β, MCP-3, IFN-γ, MCP-1, Rantes, MIP-2, IL-4, GM-CSF, IP-10, MIP-2α, IL-18A, VEGF-α, and TNF-α. Given the effect of cytokines in regulating inflammatory responses, changes in cytokine expression modes may be as potential biomarkers for DILI (Laverty et al., 2010). During the exploration of the LPS/Trovafloxacin model, neutrophil recruitment and activation, and increased cytokines such as IL-18, IFN-γ, and PAI-1 played an important role in the pathogenesis of liver damage (Shaw et al., 2009). LPS alone led to an obvious increase in the levels of IL-1, IL-6, TNF-α, CINC-1, which were remarkably amplified by co-administration with diclofenac and closely related to the observed liver injury (Ramm et al., 2015). Inflammatory stress has the potential to interact with co-exposure drug treatment and might be a susceptibility factor participated in the mechanism of IDILI (Shaw et al., 2010). As far as XLGB is concerned, a lot of patients have inflammatory-related diseases, including osteoarthritis and rheumatoid arthritis. This result is consistent with diclofenac, which is well known to induce IDILI (Kishida et al., 2012). In addition, our previous epidemiological investigations have shown that many patients with immune or inflammation-related diseases such as osteoarthritis, psoriasis, and systemic lupus are prone to cause liver injury after taking Polygoni multiflora radix (*Reynoutria multiflora* (Thunb.) Moldenke) (Zhu et al., 2016a). Accordingly, inflammatory mediators evoked by concurrent LPS exposure should alter liver homeostasis to provide the cells more susceptible to toxic chemicals in XLGB.

Inflammation is not only a physiological response to harmful stimuli but also an important factor to the pathogenesis of various metabolic disorders (Kim et al., 2017). As shown in **Figure 6B**, 12 significant metabolites accountable for class discrimination were enriched into 10 metabolic pathways. Sphingolipid metabolism, phenylalanine metabolism, and glycerophospholipid metabolism are the top 3 important pathway. Emerging experimental evidence suggests that sphingolipids can be intimately involved in inflammation (Sakamoto et al., 2019). It has been reported that LPS can increase the levels of sphinganine in serum, indicating that LPS induces the release of TNF-α and IL-1β, thereby activating a variable sphingosine kinase (Jang et al., 2007). As a second messenger molecule of sphingolipids, ceramide also acts as a systemic mediator of inflammation (Lightle et al., 2003). The production of ceramide in the liver increased the secretion of cytokines such as IL-6, TNF-α, and IL-1β, which should contribute to enhancing inflammatory responses in metabolic diseases (Schilling et al., 2013). Ceramide synthase catalyzes the N-transacylation of sphinganine to form dihydroceramides and then to ceramide in the *de novo* sphingolipid biosynthesis pathway (Tolleson et al., 1999). In the present study, the results demonstrated that sphinganine levels increased and ceramide decreased after LPS-treatment. Therefore, the concentration of sphinganine decreased in LPS group may be relevant to sphingosine accumulation. Glycerophospholipids and sphingolipids are structural units of biological membranes and act as bioactive mediators and signaling molecules in inﬂammation process (Dang et al., 2016). Glycerophosphoethanolamine (PE) is an important lipid marker of inflammation in glycerophospholipid metabolism. Increased concentrations of PE was detected in systemic circulation of Nonalcoholic steatohepatitis patients (Anjani et al., 2015). Inflammation is characterized by elevated plasma levels of PE and elevated proinflammatory cytokines IL-6, MCP-1, IL-8, and TNF-α (Ruiz et al., 2015). The results revealed that LPS dramatically altered numerous metabolic pathways that were associated with inflammatory response, immune modulation, and nutrient metabolism, which all may play a crucial part in the susceptibility of XLGB-induced liver injury.

At present, the hepatotoxic chemicals attributed to XLGB-induced liver injury are not still clarified. Epimedii folium (*Epimedium brevicornu* Maxim.) and Psoraleae fructus (*Cullen corylifolium* (L.) Medik.) could amplify inflammation cytokines such as TNF-α, IL-6, and IL-1β individually or in combination, resulting in liver injury in the LPS rat model (Gao et al., 2020). These two herbs are also contained in XLGB. Whether they are the main hepatotoxic drugs for XLGB-induced liver damage needs further study.

However, there are some limitations in this experiment. Although factors and biomarkers related to XLGB susceptibility have been explored, they still need to be validated and systematically clarified in the clinic. Additionally, the importance of mild immune stress should be validated through immunodeficiency animal models. The material basis of XLGB IDILI and its related biological mechanisms are also required for further illumination.

## Data Availability Statement

All datasets generated for this study are included in the article/**Supplementary Material**.

## Ethics Statement

The animal study was reviewed and approved by: All procedures on animals and their care complied with the Guiding Principles for the Care and Use of Laboratory Animals of China and Institutional Animal Care and Use Committee of 302 hospital of PLA. Fifth Medical Center of Chinese PLA General Hospital(formerly named as 302 hospital of PLA).

## Author Contributions

C-yL and MN performed the experiments, analyzed the data and wrote the manuscript. Y-lL, J-fT, and WC, and collected and prepared samples. GQ, M-yZ, and Y-fS performed the analyses. J-zL, X-jL, and R-sL amended the paper. X-hX, G-hL, and J-bW designed the study and amended the paper.

## Funding

This research is supported by the National Key R&D Program of China (No. 2018YFC1707000), the National Natural Science Foundation of China (Nos. 81630100, 81721002, and 81503350), CAMS Innovation Fund for Medical Sciences (CIFMS) (Grant no. 2016-I2M-1-001) and Beijing Nova Program (No. Z171100001117114).

## Conflict of Interest

The authors declare that the research was conducted in the absence of any commercial or financial relationships that could be construed as a potential conflict of interest.
