# Supplementary material for: Screening for Susceptibility-Related Factors and Biomarkers of Xianling Gubao Capsule-Induced Liver Injury
Source: Front Pharmacol. 2020 May 29;11:810. doi: 10.3389/fphar.2020.00810 (PMC7274038; doi:10.3389/fphar.2020.00810)
Supplement: Supplementary file 1 [file DataSheet_1.docx]

***Supplementary materials for:***

**Screening for** **Susceptibility-Related Factors and Biomarkers of Xianling Gubao Capsule-Induced Liver Injury^[[1]](#footnote-1)^**

***Chun-yu Li^1†^,*** ***Ming niu^2†^, Ya-lei Liu^2^, Jin-fa Tang^3^, Wei Chen^1^,*** ***Geng Qian^1^, Ming-yu Zhang^1^, Ya-fei Shi^1^, Jun-zhi, Lin^4^, Xing-jie Li^5^, Rui-sheng Li^5^, Xiao-he Xiao^2^, Guo-hui Li^1 *^,*** ***Jia-bo Wang******^2*^***

*1 National Cancer Center/National Clinical Research Center for Cancer/Cancer Hospital, Chinese Academy of Medical Sciences and Peking Union Medical College, Beijing, China, 2 China Military Institute of Chinese Medicine, Fifth Medical Center of Chinese PLA General Hospital, Beijing, China, 3 The First Afﬁliated Hospital of Henan University of Chinese Medicine, Zhengzhou, China, 4 Central Laboratory, Hospital of Chengdu University of Traditional Chinese Medicine, Chengdu, China, 5 Research Center for Clinical and Translational Medicine, Fifth Medical Center of Chinese PLA General Hospital, Beijing, China*

**1.** **Chemical compositions determination of Xianling Gubao Capsule**

**HPLC conditions**

The main consituents in th Xianling Gubao Capsule(XLGB) used in the experiment were analyzed by HPLC. HPLC was performed using an Agilent 1200 HPLC system (Agilent Technologies, USA). Chromatographic separation and detection of samples were performed on a ZORBAX Eclipse Plus C18 column (4.6mm×250mm, 5μm) at a column temperature of 35°C with a flow rate of 1 mL/min. UV detection was performed at 270nm. The mobile phase consisted of solvent A (0.1% phosphoric acid) and solvent B (acetonitrile) with a gradient elution of 0-140min, 0-5 min, 95%-95 % A; 80-95min, 64 %-52 % A; 95-110 min, 52%-20 % A; 110-112min, 20%-0% A; 112-125 min, 0%-0 % A, 125-135 min, 0%-95 % A, 135-140 min, 95%-95 % A was used. The standard solutions and XLGB extracts were filtered through a 0.22 μm Millipore membrane (Carrigtwohill, Co. Cork, Ireland) and injected into the HPLC system for chemical analysis.

**Figure S1** Chemical compositions determination of Xianling Gubao Capsule by HPLC. A. the chromatogram of Epimedin A, Epimedin B; Epimedin C, icariin, psoralen, angelicin, icarisidⅡand anhydroicaritin standard mixture. B. the HPLC profile of XLGB.

**Figure S2** Metabolomic Profile Analysis of XLGB-induced liver injury. (A)PCA score plots ofof different groups in ESI- mode; (B) cluster analysis of the 352 significantly changed ions among the normal, LPS, XLGB, and LPS/XLGB groups. The colors from blue to red indicate the relative contents of metabolites.

**Figure S3** Analysis of potential biomarkers associated with susceptibility to XLGB-induced liver injury. OPLS-DA analysis of the data generated from the LPS vs normal(A), XLGB vs normal(B) in the ESI- mode. S-score plots constructed from the supervised OPLS analysis. Metabolite ions with VIP value >1 were marked with a red square. (C)the shared and unique numbers of metabolites were also visualized in Venn diagram from LPS vs normal and XLGB vs normal.

1. * Correspondence: Jia-bo Wang, Current address: No. 100 West 4th Ring Middle Road, Fengtai District, Beijing, 100039, China. Tel.: +86 1066933323. E-mail address: pharm sci@126.com; Guo-hui Li, Current address: No. 17 Panjiayuan Street South, Chaoyang District, Beijing 100021, China. Tel.: +86 1087788577. E-mail address: tcm_sci@126.com.

   †These authors have contributed equally to this work. [↑](#footnote-ref-1)
