# Supplementary figures and images for: Screening for Susceptibility-Related Factors and Biomarkers of Xianling Gubao Capsule-Induced Liver Injury
Source: Front Pharmacol. 2020 May 29;11:810. doi: 10.3389/fphar.2020.00810 (PMC7274038; doi:10.3389/fphar.2020.00810)

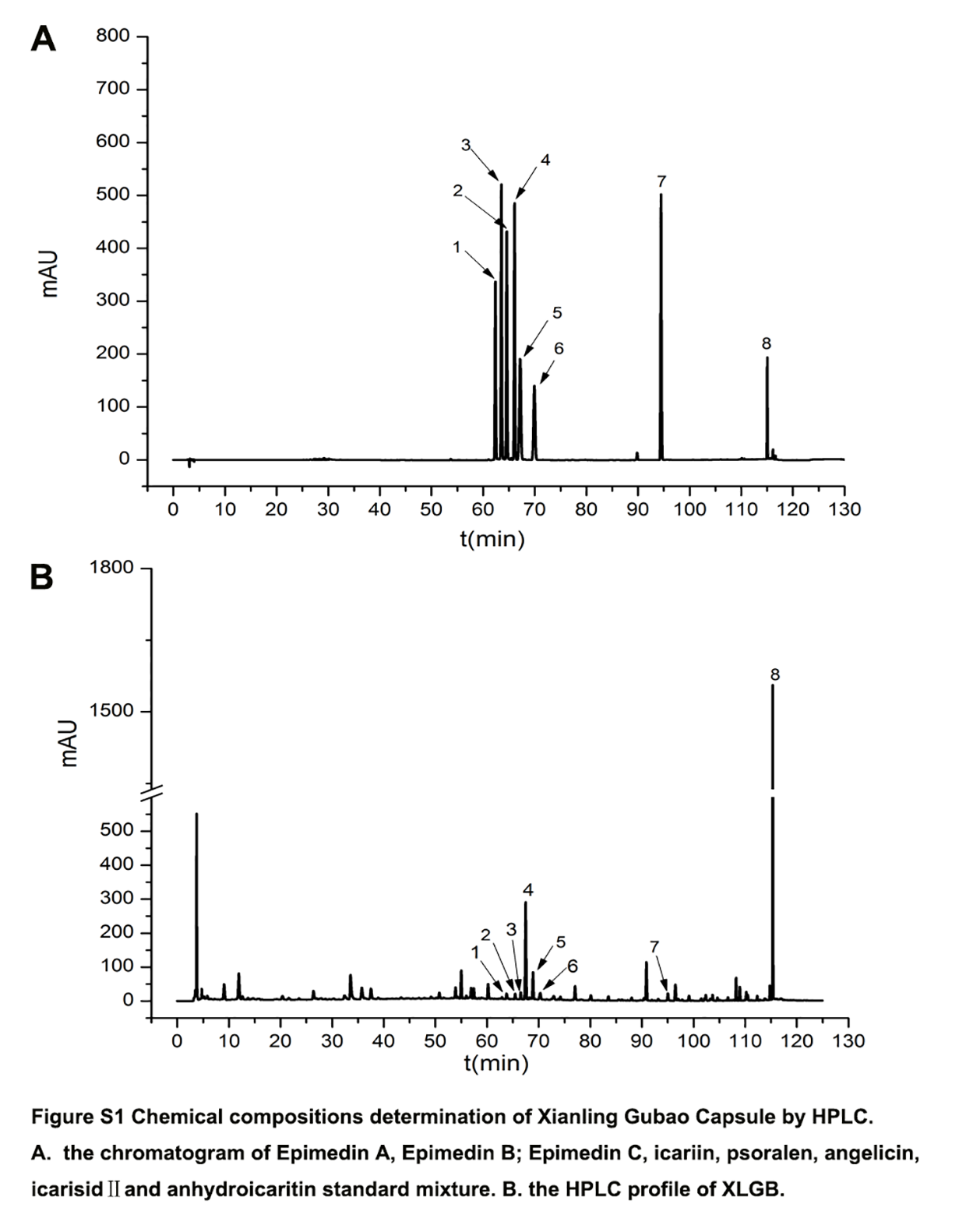

Supplement: Supplementary file 2 [file Image_1.tif]

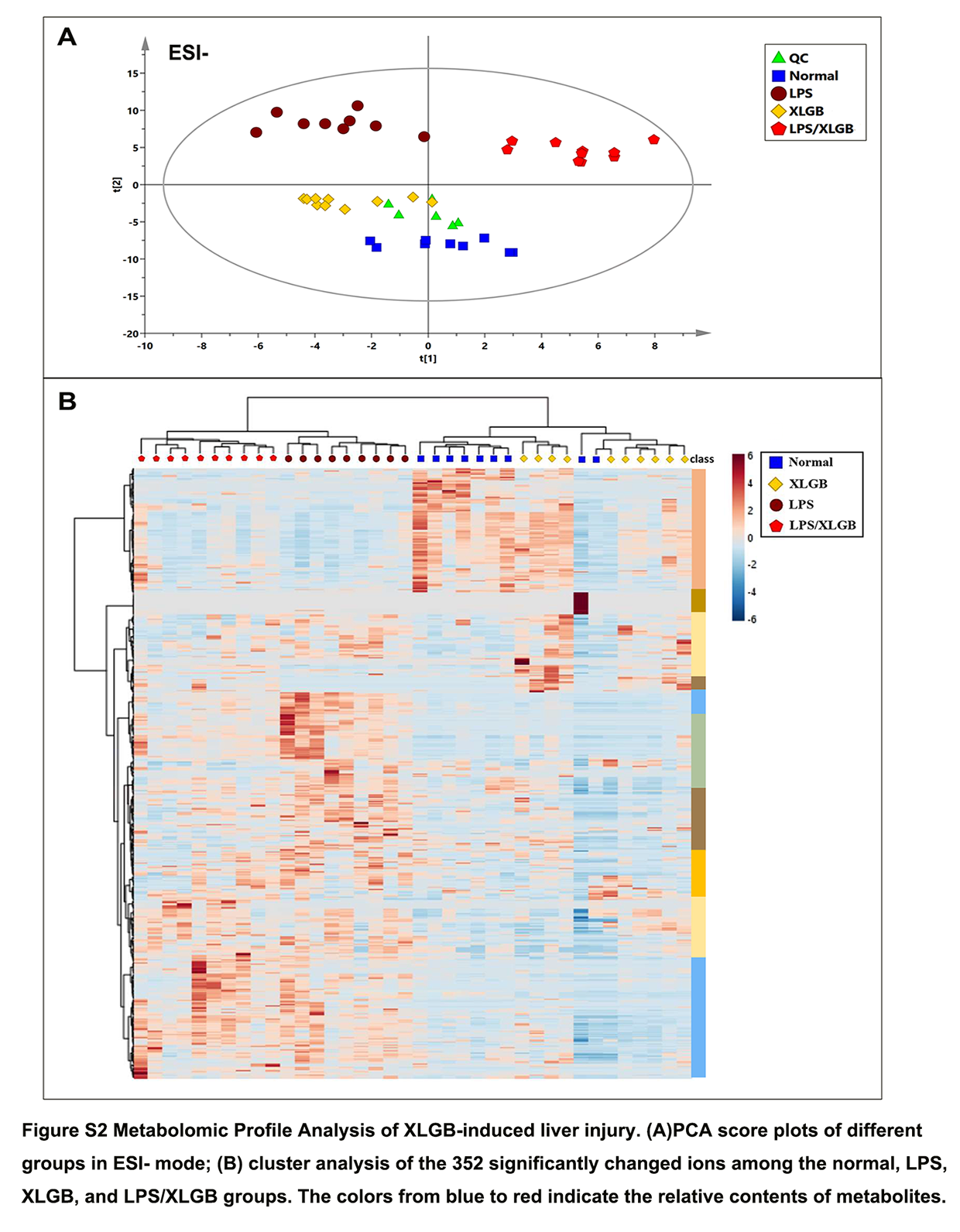

Supplement: Supplementary file 3 [file Image_2.tif]

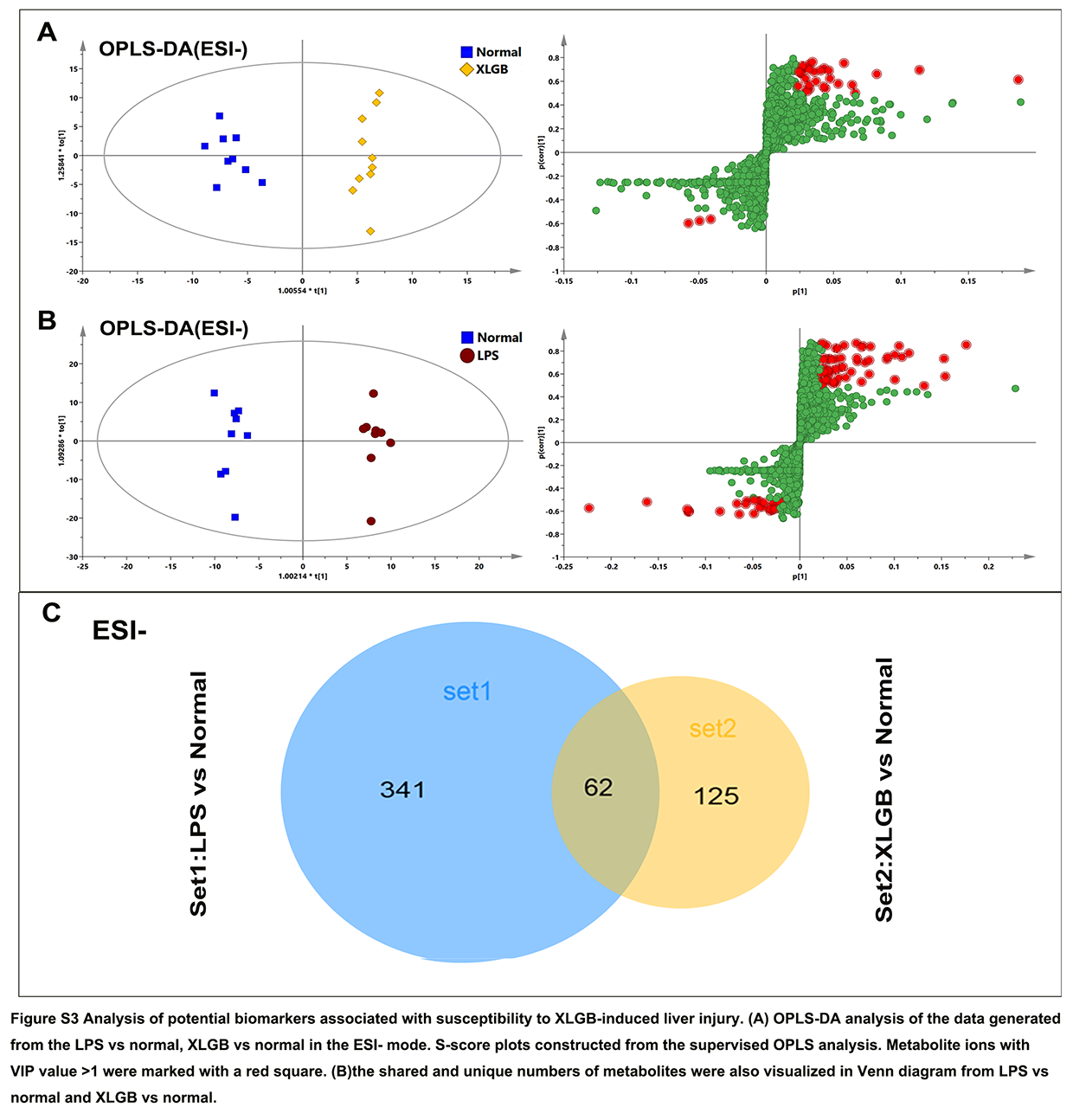

Supplement: Supplementary file 4 [file Image_3.tif]
