# Supplementary material for: Screening for Susceptibility-Related Factors and Biomarkers of Xianling Gubao Capsule-Induced Liver Injury
Source: Front Pharmacol. 2020 May 29;11:810. doi: 10.3389/fphar.2020.00810 (PMC7274038; doi:10.3389/fphar.2020.00810)
Supplement: Supplementary file 5 [file Table_1.docx]

**Table S1** Identification of potential biomarkers associated with susceptibility to XLGB-induced liver injury

| Number | Biomarkers | Formula | Mass  (m/z) | Pathway | LPS *vs* Normal | |
| --- | --- | --- | --- | --- | --- | --- |
|  |  |  |  |  | FC | P |
| 1 | Phenylalanine | C9H11NO2 | 165.079 | Phenylalanine, tyrosine and tryptophan biosynthesis | 2.445 | 1.00E-02 |
| 2 | Spermidine | C7H19N3 | 145.158 | beta-Alanine metabolism | 0.409 | 2.56E-02 |
| 3 | Calcitriol | C21H32O2 | 316.240 | Steroid biosynthesis | 6.297 | 1.70E-03 |
| 4 | Pregnenolone | C27H44O3 | 416.329 | Steroid hormone biosynthesis | 0.250 | 9.22E-03 |
| 5 | Sphinganine | C18H39NO2 | 301.298 | Sphingolipid metabolism | 3.866 | 5.67E-03 |
| 6 | Ceramide | C42H81NO3 | 647.622 | Sphingolipid metabolism | 0.444 | 5.54E-03 |
| 7 | Stearidonic acid | C18H28O2 | 276.209 | alpha-Linolenic acid metabolism | 2.027 | 2.92E-02 |
| 8 | γ-Linolenic Acid | C18H30O2 | 278.225 | Linoleic metabolism | 2.302 | 5.81E-03 |
| 9 | Glycerophosphoethanolamine | C5H14NO6P | 215.0559 | Glycerophospholipid metabolism | 3.917 | 7.23E-06 |
| 10 | 5'-Methylthioadenosine | C11H15N5O3S | 297.0896 | Cysteine and methionine metabolism | 3.058 | 3.16E-04 |
| 11 | Glycocholic acid | C26H43NO6 | 465.3090 | Primary bile acid biosynthesis | 0.372 | 3.76E-02 |
| 12 | Phosphatidylethanolamine | C41H82NO8P | 747.5778 | Glycerophospholipid metabolism | 0.427 | 1.61E-02 |
